# Impulsive dispositions and alcohol: what we know, how we know it, and where to go from here

**DOI:** 10.1186/s40479-018-0081-0

**Published:** 2018-03-09

**Authors:** Angela K. Stevens, Brittany E. Blanchard, Andrew K. Littlefield

**Affiliations:** Department of Psychological Sciences Texas Tech University, MS 2051 Psychological Sciences Building, Rm. 404, Lubbock, Texas 79409-2051 USA

**Keywords:** Impulsivity, Alcohol, Alcohol use disorder, Assessment, Self-report, Laboratory-based tasks

## Abstract

**Background:**

Relations between impulsigenic traits and alcohol-related outcomes have been the focus of much research, yet precise relations remain elusive. Historically, research used broadband conceptualizations of impulsivity, which yielded inconclusive findings. Attempts to ameliorate this problem led to more work on narrowband assessments of impulsivity. Despite that several narrowband self-report measures exist, few demonstrate adequate psychometric properties. Given the limits of self-report, researchers have also utilized laboratory-based measures of impulsive dispositions; however, this seems to have contributed more uncertainty to the literature.

**Review:**

We review commonly used self-report and laboratory-based measures of narrowband impulsivity, as well as assessments of alcohol-related constructs (e.g., consumption and consequences). We discuss remaining issues in impulsivity and alcohol assessment, which limit understanding of how impulsigenic traits influence alcohol-related behaviors. Cutting-edge conceptualizations and assessment of state-level impulsivity are also discussed.

**Conclusions:**

More work is necessary to further this area of research, including establishing consistent nomenclature and a cohesive conceptualization of impulsigenic traits as they relate to alcohol use and alcohol use disorders.

## Background

Impulsigenic traits are transdiagnostic, as “impulsivity” is a symptom criterion for several psychological disorders in the *Diagnostic and Statistical Manual of Mental Disorders*, Fifth Edition (*DSM*-5), including borderline personality disorder and attention-deficit/hyperactivity disorder [1]. In addition to being included in specific criteria sets for some disorders, impulsivity-like traits are thought to be etiologically relevant to several disorders, including substance use disorders. Indeed, some of the most robust personality predictors of alcohol use and related problems are impulsigenic traits [2–4] (see Littlefield & Sher [5] for more details). A multitude of definitions and assessments of “impulsivity” have been used in the literature to link these traits to several alcohol-related constructs (e.g., various indices of alcohol use, problems, and disordered drinking). The purpose of this article is to review and synthesize conceptualizations and assessments of impulsivity and alcohol-related constructs. Strengths and limitations of relevant literatures are summarized. Further, relations between impulsigenic traits and problematic alcohol use among adults are reviewed in the context of the conceptual, methodological, and analytical limitations of the extant literature. Finally, suggestions for future research are provided.

## Conceptualization and assessment of impulsivity

Impulsigenic traits have garnered significant attention in the literature given their relevance to psychopathology (see Berg, Latzman, Bliwise, & Lilienfeld [6] and Sharma, Markon, Clark [7]). Although impulsivity may be an etiologically important construct contributing to pathological alcohol use (and other psychological conditions), research progress remains somewhat hampered by inconsistencies in conceptualizations of impulsivity. Broadband impulsivity has historically been ill-defined, which has contributed to a muddled literature (see Evenden [8] and Cyders [9]). In fact, Block [10] describes a critical flaw of impulsivity assessment (i.e., using broadband, heterogeneous measures) using the “jingle” (i.e., two constructs with similar labels are distinctly different) and “jangle” (i.e., two constructs with different labels are equivalent constructs) fallacies. Initially, “impulsivity” was measured as part of comprehensive personality assessments (e.g., constraint subscale of Tellegen’s three-factor model, psychoticism subscale of Eysenck and Eysenck’s three-factor model, impulsive-sensation seeking subscale of Zuckerman’s alternative five-factor model [11–13]). Later, behavioral tasks (e.g., continuous performance tasks) purported to assess “impulsivity” became more common. As a result, the existing literature is riddled with various references to “impulsivity” though multiple assessments and definitions have been used to define a number of theoretically discrete constructs (see Evenden [8]). Further, given recent calls to assess homogenous constructs [14], some consider the term “impulsivity” to be inaccurate ([4]; see Cyders [9]), and recent research has emphasized a “splitting” (rather than “lumping”) approach to assessment (e.g., Blanchard et al. [15]). In addition to an enhanced focus on types of impulsivity, there has also been an increased interest in designing measures to distinguish trait- and state-level impulsivity (e.g., Tomko et al., [16]), which adds an exciting intricacy to this enigmatic literature.

## Self-report assessment of impulsive dispositions

Although there are a multitude of self-report assessments that purport to assess various types of impulsivity (e.g., see Sharma, Kohl, Morgan, & Clark [17]; the discussion of Reise, Moore, Sabb, Brown, & London [18]), we focus on two of the most widely used scales: the Barrett Impulsiveness Scale (BIS-11) [19] and the UPPS-P Impulsive Behavior Scale [20]. The BIS-11 is comprised of three domains (i.e., motor, nonplanning, and attentional impulsivity) with six facets each. Despite that a strength of this assessment is measurement of impulsivity-like traits, researchers often report a total score when using the BIS-11, which assumes impulsivity to be a unidimensional construct (see Stanford et al. [21]). Although frequently used, rigorous psychometric assessment of the BIS-11 is limited; however, recent research suggests suboptimal fit for the unidimensional, bifactor, six correlated-factors, and second-order factors models (see Reise, et al. [18]). Further, in a large adult sample, internal consistency was poor-to-acceptable (α = .59-.74) for the three domains and unacceptable-to-acceptable for the six facets (α = .27-.72) [21]. Test re-test reliabilities at one-month were also substandard across domains (*r* = .61-.72) and facets (*r* = .23-.74).

Derived from the five-factor model of personality [22, 23], another commonly-used measure of impulsivity-like facets is the UPPS-P [20]. The UPPS-P measures the following five narrowband impulsivity-like traits: 1) sensation seeking, or the tendency to engage in new and thrilling experiences, 2) lack of planning, or the tendency to act without thinking, 3) lack of perseverance, or the inability to focus attention on a difficult or boring task, 4) positive urgency, or the tendency to act rashly under extreme positive mood, and 5) negative urgency, or the tendency to act rashly under extreme negative mood. The UPPS-P consistently demonstrates strong psychometric properties, including acceptable-to-excellent internal consistency (e.g., .83 to .94 across subscales among college students) [22] and high test-retest reliabilities in a nonclinical emerging adult sample (e.g., .81 to .93 across subscales) [24]. Further, tests of measurement invariance indicate the UPPS-P is invariant across Hispanic and non-Hispanic students [25]. Although additional work is warranted, evidence also indicates the UPPS-P may be invariant across gender [26]. As a result, the UPPS-P has received endorsement from the National Institutes of Health’s (NIH) PhenX Toolkit [27] as the recommended self-report measure of impulsigenic traits.

Unlike the BIS-11, researchers tend to report facet-level scores of the UPPS-P (rather than an overall score), which utilizes the multidimensionality of this scale and is likely a more accurate reflection of impulsigenic trait structure, more broadly. Illustrating this issue, using principal components analysis, BIS subscales loaded onto multiple narrowband UPPS facets, indicating BIS subscales may represent heterogenous constructs and/or items (see Whiteside & Lynam [23]).

Although there are distinct differences among traditional conceptualizations of impulsivity (as noted above), these models of assessment are similar in that impulsivity is conceptualized as a comparatively stable trait. Indeed, this trait conceptualization provides information regarding individuals’ predispositions for impulsigenic behavior, though clinically relevant information is missing. More specifically, trait impulsivity assessments do not identify *when* an impulsive behavior will occur – or state impulsivity (see Tomko et al. [16]).

Most recently, Tomko et al. [16] developed a self-report measure of momentary impulsivity (i.e., Momentary Impulsivity Scale [MIS]), which is the first self-report measure of state-like impulsivity. Prior to the development of the MIS, state impulsivity has been ostensibly examined via laboratory-based behavioral tasks, as these tasks provide “behavioral snap shots” of how individuals would respond in a particular situation (see Cyders & Coskunpinar [28], p. 967). In this growing area of research, the introduction of the MIS offers the field a more viable option for rigorous psychometric research, compared to laboratory-based tasks, to improve our ability to accurately assess state-level impulsivity. The between- and within-person one-factor structure of the MIS exhibited good fit to the data, and the scale demonstrated high between-person reliability (or rank ordering of individuals remained stable across time) and moderate within-person reliability, which provided evidence for its state-like properties [16]. Further, Tomko et al. [16] also provided preliminary evidence for content validity of the MIS, as it significantly correlated with three of the four UPPS facets (i.e., urgency, lack of planning, and lack of perseverance) and the three BIS subscales and total score in the overall sample. In addition to using the MIS, other approaches to examine within-person impulsivity over time include the use of psychometrically-validated trait measures of impulsivity (e.g., the UPPS; [23] with EMA [29]).

## Assessment of impulsive dispositions via laboratory-based behavioral tasks

Although self-reported assessments of impulsivity have multiple strengths (e.g., relative ease of administration; detailed psychometric evaluations of some scales), there are also notable limitations to self-report measures (e.g., social desirability bias, face validity; see Northrup [30] for self-report limitations and additional discussion in *Strengths and Limitations of Impulsive Disposition Assessment* below). In part to address these limitations, laboratory-based tasks remain popular behavioral indices of impulsivity. Similar to the self-report assessment literature, research examining laboratory-based behavioral tasks of impulsigenic traits is complicated by the existence of numerous tasks purported to assess distinct facets of impulsivity (e.g., response inhibition vs. delay discounting; see Cyders & Coskunpinar [28] and see Dick et al. [31]). Laboratory-based tasks often assess multiple forms of “impulsivity,” including behavioral undercontrol and attentional processes (see Hamilton et al. [32, 33]). For example, response inhibition tasks, such as the Go-Stop paradigm (see Dougherty, Mathias, Marsh, & Jagar [34]), require inhibition of motor responses when signaled by the changing of a stimulus. Similarly, the Immediate and Delayed Memory tasks (IMT/DMT) assess rapid response impulsivity [35]. Another behavioral task is the continuous performance task [36], which assesses response inhibition, as well as initiation and attention. Further, another attentional indicator of “impulsivity” is distortions in time perception (e.g., Time Paradigm) [34]. Another distinct, though important construct, often assessed behaviorally (c.f., Monetary Choice Questionnaire [MCQ]) [37], is delay discounting, or the preference for smaller, more immediate rewards (e.g., Two Choice Impulsivity Paradigm [TCIP], Single Key Impulsivity Paradigm [SKIP]) [34]. Efforts to examine psychometric properties of laboratory-based behavioral tasks (e.g., test-retest reliability) suggest variability in reliability across task types. For example, in a sample of healthy adults assessed on average approximately nine days apart, test-retest reliability varied across tasks: inattention (including CPT omission errors; *r* = .38-.42), impulsive action (as measured by the stop signal task, Go/NoGo task, and CPT commission errors; *r* = .65-.73), and impulsive choice (including delay discounting; *r* = .76-.89) [24]. See Fillmore and Weafer [38] for an overview of laboratory-based behavioral tasks, including strengths and limitations of several behavioral tasks.

## Relations between self-report and laboratory-based tasks

Efforts to bridge the gap between the self-report and laboratory task literature have utilized advanced statistical approaches (e.g., meta-analytic, structural equation modeling) to conceptualize the latent structure of impulsivity, and often include the UPPS framework. For example, Sharma et al. [7] used a meta-analytic approach to capture the structure of impulsivity. These findings indicated “impulsivity” consists of four distinct impulsigenic traits (i.e., the four traits assessed by the UPPS) and four behavioral/cognitive impulsivity-related constructs (i.e., inattention, inhibition, impulsive decision-making, and shifting). Similarly, MacKillop et al. [39] used a combination of self-report (i.e., BIS-11, UPPS-P, MCQ) and laboratory-based tasks (i.e., Go/NoGo Task, Conner’s CPT) to assess a proposed latent structure of impulsivity comprised of three distinct domains: impulsive choice (i.e., inability to delay monetary gratification), impulsive action (i.e., response inhibition failure), and impulsive personality traits (e.g., attention, nonplanning, lack of perseverance). Although sensation seeking was tested, it did not load onto the impulsive personality domain (i.e., λ < .2). With sensation seeking removed, they achieved adequate fit for the three-factor model; however, this solution largely reflected method effects (e.g., all of the self-reported assessments, including the various facets of the UPPS, loaded onto the same “impulsive personality” trait). Consistent with these findings, in a meta-analysis by Cyders and Coskupinar [28], the mean sample-size weighted effect size between behavioral tasks and UPPS-P self-report was small (*r* = .10). More specifically, lack of perseverance, lack of planning, and negative urgency were associated with failure to inhibit prepotent response (*r* = .10, *r* = .11, and *r* = .11, respectively). Lack of planning was also linked to delay discounting (*r* = .13) and distortions in response time (*r* = .10), whereas sensation seeking was only related to delay discounting (*r* = .06). In a separate study, negative urgency was correlated with shorter delay latency on the TCIP (*r* = -.29), and sensation seeking was linked to distortions in elapsed time (*r* = -.23) [40]; notably, in another study [41], the magnitude of the correlation between negative urgency and TCIP was higher than the correlation (*r* = .14) between self-report delay discounting (as assessed by the MCQ [37] and laboratory-based delay discounting (as assessed by the TCIP) [34]. Evidence also suggests that BIS-11 domains and facets were uncorrelated with IMT, DMT, GoStop, TCIP, and SKIP (see Stanford et al. [21]). These findings suggest that prepotent response inhibition failure corresponds most consistently with self-reported impulsigenic traits; however, it is evident that self-report and laboratory-assessed impulsivity appear to assess distinct constructs with little shared variance (see Cyders & Coskunpinar [28]).

## Strengths and limitations of impulsive disposition assessment

### Self-report assessments

Broadly, strengths of self-report assessment include their cost-effectiveness, efficiency, ease of dissemination, and face validity. That said, there are notable limitations to self-report, including face validity (e.g., participants may not be motivated to respond in a honest manner; see Cyders & Coskunpinar [28] for more details). More specific to “impulsivity,” Reise et al. [18] noted multiple issues with the BIS-11, including the following: “(a) low or near-zero correlations of some items with others; (b) highly redundant content of numerous item pairs; (c) items with salient cross-loadings in multidimensional solutions; and ultimately; (d) poor fit to confirmatory models”; moreover, they conclude, “use of the BIS-11 total score as reflecting individual differences on a common dimension of impulsivity presents challenges in interpretation” (p. 631).

Even among the “gold standard” of self-report assessment, some are reconsidering the utility of splitting urgency (i.e., combining positive and negative urgency to reflect an overall affective urgency; [42–44] to combat potential redundancy or suppressor effects in multivariate models. As a recent recommendation notes,

It is important to appreciate that the two urgency traits correlate highly with each other, with correlation values ranging from .46 (Cyders and Smith, 2007) to .69 (Settles et al., 2014). For that reason, when the two traits do not predict differently (which may be the case in the prediction of problem drinking or drug use), it may be wise to combine them and use the overall urgency trait. (Smith & Cyders, [45], p. S7).

Further, though there is some initial evidence of measurement invariance of the UPPS-P across gender [26], additional work could examine the impact of assumptions regarding indicator scaling (i.e., specifying items as continuous versus categorical). Beyond psychometric issues, others have criticized the UPPS impulsivity framework on theoretical grounds (see Gullo, Loxton, & Dawe [46]). Clearly, a consensus on the conceptualization of impulsigenic traits has not been reached, even among developers of the scale (e.g., [42–44, 47]).

Another approach to impulsivity assessment is the “lumping” of various subscales to create idiosyncratic, heterogeneous assessments of “impulsivity.” This approach can lead to both psychometric and interpretational concerns. Demonstrating this issue, previous work examining “behavioral undercontrol” utilized subscales from multiple assessments, which may or may not reflect aspects of impulsive behavior (i.e., the Novelty Seeking scale of the Tridimensional Personality Questionnaire [TPQ] [48], the Psychoticism subscale of the Eysenck Personality Questionnaire-Revised [EPQ-R] [12], and the reverse-scored Lie subscale of the EPQ-R) [49]. Approaches which lump multiple measures may yield different substantive findings, limit comparability across studies, and impede and meta-analytic endeavors.

### Laboratory-based behavioral tasks

Laboratory-based tasks are thought to address some of the limitations of self-reported assessments. Indeed, these methods are purported to measure individuals’ behaviors, as opposed to how individuals think they would respond in a given situation (see Cyders & Coskunpinar [28]). However, one primary concern of behavioral tasks is the limited ecological validity and the use of different tasks (as well as inherently different conceptualizations) to measure similar constructs, which precludes researchers from making accurate comparisons across studies (see King Patock-Peckham, Dager, Thimm, & Gates [50] and see Sharma et al. [7]).

For example, given laboratory tasks are capturing a specific behavior within a discrete period, it is argued these tasks are more reflective of state-level (as opposed to trait-level) impulsivity [28, 40]. Despite this, evidence suggests moderate-to-high test-retest reliability for a number of these tasks, suggesting more trait-like, rather than state-like, qualities (see Weafer et al. [24]). Laboratory-based assessments also have different parameters that can be altered by researchers, and these are often not made explicit in research using such assessments. For instance, researchers can change the percentage of stop trials on the Stop-Signal Reaction Time Task (SSRT), which can impact correlations with self-report measure of impulsigenic traits ([51–53]; see Sharma et al., [7]). Moreover, the tasks purported to measure the same dimensions of “impulsivity” (e.g., inhibition) demonstrate weak-to-nonexistent correlations (see Rey-Mermet et al. [54]). For other limitations of using laboratory-based tasks to measure individual differences, see Hedge, Powell, and Sumner [55].

Moreover, although impulsivity assessment using multi-trait, multi-method (MTMM) approaches have been executed (e.g., Smith et al. [4]; MacKillop et al. [39]), more work is needed. Specifically, in Smith et al. [4], self-report assessments of the UPPS-P were compared to orally-administered assessments of the same scale. One major reason to utilize a MTMM approach is to reduce method variance (e.g., self-report assessments may show overlap due to response bias related to social desirability); however, the use of orally-administered UPPS-P items do not quell the limitations of self-administered self-report items (e.g., response bias). Indeed, this approach may increase bias due to social desirability pressures [56]. Thus, this type of work may not reflect a true MTMM approach in the traditional sense [57]. More traditional MTMM approaches have been used (i.e., include self-report and laboratory tasks) [39]. However, as noted previously, these findings should be interpreted with caution, as it appears some solutions reflect method variance (i.e., in MacKillop et al. [39] all self-reported impulsivity measures loaded onto the same factor despite the notion that these measures purportedly assess multiple, distinct constructs) rather than the identification of latent constructs. Without understanding and appropriately modeling the true latent structure of impulsive dispositions, we can continue to expect inconsistent, and, at times, puzzling findings.

## Conceptualization and assessment of alcohol-related outcomes

As with impulsivity, establishing consistent operational definitions and terminology for alcohol-related outcomes is crucial if one seeks to understand the “impulsivity-alcohol” relation. Much debate remains regarding the classification of consumption, alcohol-related problems, and AUDs. For example, under the previous classification system, alcohol abuse and alcohol dependence were differentiated, though this distinction has been replaced by alcohol use disorder in the *DSM-5* [1]. Although this change includes many improvements (e.g., removing of legal issues, addition of craving) [58] and may improve diagnostic validity and reliability by reducing diagnostic imposters (see Lane & Sher [59]), the new criteria are not without limitations. Specific issues remaining include treatment of symptoms as equivalent despite varying degrees of severity (e.g., tolerance versus withdrawal; [60]), disregard for symptom patterns [59], and use of consequences in establishing diagnoses (see Martin, Chung, Kirisci, & Langenbucher [60]). Additionally, emerging work based on Item Response Theory (IRT) indicates substantial variability in the difficulties (closely related to base rates) of AUD symptoms as a function of the instrument used for assessment (see Lane, Steinley, & Sher [61]), which creates challenges for work focused on linking impulsivity-like traits with specific symptoms of AUD.

## Assessment of alcohol use and alcohol-related consequences

It is important to note that though the assessment of consumption is not currently included as criteria for an AUD (though this has been considered, e.g., Hasin et al. [58]), alcohol consumption is necessary to meet criteria for AUD. To assess consumption, researchers and clinicians have several self-report measures from which they can choose, though other indices are now available (e.g., biomarkers; see *Summary and Future Directions*). For example, many use quantity-frequency (Q-F) items, which typically assess various indices of consumption (e.g., daily quantity, quantity of greatest consumption, average frequency, frequency of binging) over a specified amount of time. These measures can then be used to create Q-F scores [62, 63] or items can be used individually as separate outcome measures. More standardized forms include the Timeline Followback Procedure (TLFB), which has evidence for acceptable psychometric properties [62, 64] and the Daily Drinking Questionnaire-Revised (DDQ-R) adapted from the original DDQ [65]. For example, the DDQ-R asks individuals to estimate the number of standard drinks consumed in a typical week from the past month. There are also various indices of “risky drinking.” For example, to quantify so-called binge drinking, the National Institute of Alcohol Abuse and Alcoholism’s (NIAAA) conceptualization, defined as 4+ drinks in a two-hour period (5+ for males), is increasingly becoming the accepted definition. Despite this improvement, several terms are used seemingly interchangeably in the literature, (e.g., problematic drinking, excessive drinking, heavy episodic drinking), which exacerbates conceptualization and assessment issues.

Dozens of assessments of alcohol-related consequences exist, and commonly used measures include screeners like the Alcohol Use Disorder Identification Test (AUDIT) [66]. The AUDIT (which also includes assessments of alcohol use) exhibits good-to-excellent internal consistency reliability, with Cronbach’s alphas ranging from .77 to .94 across a variety of samples (e.g., primary care patients, college students; Allen, Litten, Fertig, & Babor [67]; see de Meneses-Gaya et al. [68] for a review of psychometric properties). More comprehensive measures of consequences, such as the Young Adult Alcohol Consequences Questionnaire (YAACQ) [69], the Young Adult Alcohol Problems Screening Test (YAAPST) [70], and the Rutgers Alcohol Problem Index (RAPI; see Neal, Corbin, & Fromme, 2006 for an improved version [71, 72]), also have evidence for acceptable psychometric properties. These measures typically assess a range of problems, including physical, intrapersonal, social, and occupational consequences. Although many of these measures include *DSM*-5 AUD criteria [1], limitations remain, including limitations inherent to self-report, as well as more alcohol-specific issues [59]. Additional issues remain in analytic approaches. For example, many researchers use a summed-score approach to consequences, which does not consider that some consequences (e.g., withdrawal) are more severe than others (e.g., hangover). Moreover, many researchers often adjust for alcohol consumption when assessing consequences as an outcome, which may create interpretational issues and result in unnecessarily adjusting relevant variance in the dependent variable [73, 74]. In sum, a consensus regarding how to define, assess, and analyze alcohol-related outcomes has yet to be reached.

## Relations between impulsive dispositions and alcohol-related outcomes

Despite the limitations regarding conceptualization and assessments of the constructs of interest, a myriad of research has examined the relations between “impulsivity” and alcohol outcomes. In most research, the methods previously reviewed (i.e., self-report and lab-based tasks of impulsivity, self-reported alcohol outcomes) are typically used to assess impulsivity-alcohol relations. However, another area of importance are alcohol-challenge studies in which individuals consume alcohol and then perform laboratory-based behavioral tasks of impulsivity. Although outside of the scope of this review, see Littlefield, Stevens, and Sher [75] for a review of developmental processes of “impulsivity” and alcohol (e.g., “maturing out”) [76], as well as other etiological models of alcohol involvement.

## Self-reported impulsive dispositions and alcohol

Regarding self-report assessment of impulsigenic traits, the BIS-11 total score is associated with alcohol consumption and use status [77, 78], as well as related problems [79–81], including early-onset AUD symptomatology [82, 83]. For example, in one study examining past-month drinking among college students, the BIS-11 total was positively associated with drinks per drinking occasion (*r* = .21) and length of drinking occasion (*r* = .14); at the subscale level, motor (*r* = .22) and cognitive subscales (*r* = .18) were associated with drinks per occasion, and cognitive was related to length of occasion (*r* = .16). Nonplanning was not associated with any index of alcohol consumption [84]. When examining UPPS-P facet-level relations and alcohol constructs, more work has been done relative to the BIS. For example, meta-analytic approaches examining mean effect sizes (ES) indicate sensation seeking is robustly associated with increased drinking frequency (*ES* = .22) and binge drinking (*ES* = .36), whereas lack of planning tends to be associated with increased drinking frequency (*ES* = .21), and alcohol-related problems (*ES* = .26) [85]. Lack of perseverance is linked to increased drinking quantity (*ES* = .32) and frequency (.28), and may be associated with drinking onset, whereas negative urgency is often associated with drinking frequency (*ES* = .22), alcohol-related problems (*ES* = .34), and AUD symptomatology (*ES* = .38) [85]. Although less work has been done with positive urgency, existing findings indicated relations with alcohol-related problems (*r* = .34; see Coskunpinar, Dir, & Cyders [85] for a meta-analysis and see Littlefield et al. [75] for a review). Further, self-reports of state-level impulsivity and it relations to alcohol-related outcomes remains in its nascent stages; however, using ecological momentary assessment (EMA), impulsivity (as assessed by the MIS) was positively associated with alcohol use at the momentary level (i.e., on a particular occasion) and at the daily level [86].

## Laboratory-based tasks and alcohol-related constructs

Typically, effect sizes for relations between laboratory tasks of impulsigenic traits and alcohol outcomes are small. In a recent meta-analysis, weighted relations of laboratory tasks and self-reported alcohol use, broadly, were small-to-medium (Go/No Go Task *r* = .18; [SSRT] *r* = .17; hypothetical delay discounting *r* = .09), except for the Iowa Gambling Task (reflecting inhibitory dyscontrol; *r* = .41) and the Stroop Color-Word Test (reflecting inattention; *r* = .41) [7]. Likewise, women who reported early-onset drinking (<18 years) compared to late-onset (>21 years) made more commissions errors on the IMT and DMT [40]. Age at first drink was also significantly negatively correlated with more impulsive responding on the DMT among women [87]. However, Rubio et al. [81] used the Continuous Performance Test (CPT) to assess commission errors, which is analogous to the IMT (see Dougherty, Bjork, Marsh, & Moeller [88]), and found no significant difference in commission errors between non-dependent, heavy drinkers (as defined by the researchers) and control participants. Using a laboratory-based hypothetical choice task, Kollins [89] examined delay discounting in a sample of college students. Earlier onset of alcohol use was associated with a preference for smaller, immediate hypothetical rewards [89]. Delay discounting was also strongly linked to “passing out” from alcohol consumption (*r* = .73) [89]. Combining self-report and laboratory-based tasks (i.e., an MTMM approach), MacKillop et al. [39] used a multivariate structural equation model and demonstrated differential relations across impulsivity-like trait and AUDIT scores. Specifically, impulsive choice, impulsive personality traits, and sensation seeking latent variables were significantly positively predictive of AUDIT scores, whereas the construct of impulsive action was unrelated (correlations not provided).

## Alcohol challenge studies and impulsive dispositions

Alcohol challenge studies are another approach to examine the impulsivity-alcohol relation. In these studies, experimentally controlled alcohol use is typically treated as the independent variable to determine its influence on behavioral task performance. These studies eliminate some limitations inherent to self-report methods and may yield more causal inferences.

For example, in some alcohol administration studies, individuals who consumed alcohol tended to discount smaller, more immediate hypothetical rewards at lower rates than sober individuals [90]. This is contrary to later findings by Dougherty, Marsh-Richard, Hatzis, Nouvion, and Mathias [91] who investigated the dose-dependent effects of alcohol on three laboratory-based impulsivity tasks (IMT, GoStop, and SKIP). Their results suggested a dose-dependent relation for commission errors on the IMT across time, whereas performance on the GoStop (a measure of response inhibition), but not the SKIP (a measure of delay discounting). Indeed, individuals responded more impulsively on the GoStop task across all time points (i.e., 0.25-hour, 1-hour, and 2-hour), regardless of dose. Alcohol consumption resulted in more delay discounting at the one- and two-hour time points, regardless of dose, on the SKIP. In sum, it appears the studies of impulsivity-alcohol relations yield equivocal findings, which may vary as a function of the task used (see Weafer & Fillmore [92] for a review).

## Summary and future directions

Although notable methodological advances have been made in the area of impulsivity and alcohol research (e.g., sophisticated frameworks of impulsigenic personality traits, advanced statistical approaches, psychometrically-supported state-level measures, alcohol-challenge studies, MTMM designs), much work is needed to elucidate relations between impulsive dispositions and alcohol-related outcomes. Research aiming to establish a conceptual model of impulsivity that integrates self-report and laboratory-based constructs is worthy of attention, as this would advance the field by increasing interpretability of findings and facilitating comparability across studies. The studies reviewed represent a necessary and important first step in this process. We now provide some notable limitations, as well as potential solutions and associated future directions we hope will advance the understanding of the impulsive disposition-alcohol relation.

One concern is the possibility that self-report and laboratory-based tasks are conceptually distinct constructs. More specifically, it is arguable that laboratory tasks are a measure of “ability” as opposed to a “response style,” and modest correlations are typical for ability vs. response style measures (see Sharma et al. [7]). If this is the case, one logical conclusion is that “the two methodologies assess different phenomena entirely – a large-scale version of the jingle phenomena – such that it is a fruitless effort to pursue any integration of these literatures” (Sharma et al. [7], p. 388). Thus, a unifying conceptualization of impulsigenic traits is needed.

We agree with Cyders [9], who asserts that if researchers continue to use the term “impulsivity” to refer to several related, but distinct constructs “we will continue to muddy the water, mask existing effects, misunderstand existing research, and fail to move forward past the question of *Is impulsivity related to psychopathology and how?*” (p. 2). Plainly stated, we caution the reader from using the term “impulsivity.”

Further, distinguishing between state- and trait-level impulsivity is an important consideration when examining alcohol use and related problems, as it is arguable that *when* an impulsive behavior occurs (i.e., state-level) is equally (or perhaps more) clinically relevant than *if* a person has the proclivity for impulsive behavior (i.e., trait-level). Assessment of state-level impulsivity is a burgeoning area of research, and future directions including examination of the MIS factor structure (outside of its original sample), convergent and discriminant validity using laboratory-based tasks (i.e., an MTMM approach), as well as investigating its criterion validity (e.g., alcohol consumption, risky behavior).

One obstacle we continue to face as we attempt to bridge the gap between self-report and laboratory-based findings is the confounding impact of method variance. Indeed, previous attempts to examine self-report and laboratory-based impulsivity measures simultaneously resulted in method components, aptly named by Meda et al. [93]. Current research attempting to construct a comprehensive conceptual model of impulsivity [39] may be confounded by method effects. Therefore, future directions include creating and/or refining laboratory-based and self-report assessments of distinct impulsigenic constructs (e.g., sensation seeking, urgency, impulsive decision-making) to be able to utilize a true MTMM approach [57]. It may also be beneficial to utilize more nuanced classifications of impulsive dispositions measured by laboratory tasks (e.g., separating impulsive decision making, motor impulsivity, and cognitive impulsivity; [94]). Additionally, measuring domain-specific impulsivity may have clinical and practical utility (e.g., the Domain-Specific Risk-Taking Scale [DOSPERT], which include areas like safety/health, recreational, and social decisions; [95]). This domain-specific approach may also be helpful in designing laboratory-based tasks to correspond to self-report measures of specific impulsive dispositions.

Further, we believe some considerations may be useful for future research utilizing existing measures. For example, when using the UPPS-P, items should be modeled as ordinal, as a 4-point Likert-type response scale for individual items does not reflect a continuous variable. Additionally, although work examining latent structures of impulsive traits use advanced methods and multimethod approaches, exploratory factor analyses (EFAs) are conducted using suboptimal methods (e.g., principal components analysis; Sharma et al., [17]), or are not conducted prior to confirmatory factor analyses [39]). For example, although the motor subscale of the BIS-11 was modeled as an impulsive personality trait, this may be a self-report measure of impulsive action [39], which may have been evidenced by appropriate exploratory models. Moreover, replication studies are needed to confirm purported conceptual models of impulsivity.

In line with current trends in impulsivity assessment, incorporating EMA designs when assessing alcohol use and associated variables (e.g., consequences, motives, and protective behavioral strategies) will also serve to further research on the impulsive trait-alcohol link (see Trull & Ebner-Priemer [96]). To utilize benefits of an MTMM approach, alcohol research endeavors can also use transdermal alcohol monitoring (e.g., Secure Continuous Remote Alcohol Monitor [SCRAM]) [97], which would also be a great improvement over tradition self-report methods. Clinically, just-in-time adaptive interventions [98] may benefit from inclusion of state-level impulsivity in algorithms for delivering interventions. Going forward, it will also be necessary for clinicians and researchers to use consistent and psychometrically-supported definitions and assessments of alcohol consumption and AUDs, as well as impulsive dispositions. To evaluate these measures and better understand relations between impulsigenic traits and alcohol-related outcomes, cognitive interviewing and observational data may be useful (see Durbin & Hicks [99]).

## Conclusions

Although multiple associations have been identified between various types of impulsive dispositions and alcohol-related outcomes, advancements in conceptualization, assessment, and methodology are necessary before a clearer understanding of these relations can be obtained. Research efforts have made great strides toward examining these complex relations, though much more is needed to discern the role of impulsigenic traits on alcohol use and related outcomes to better inform prevention and treatment of alcohol use problems and disorders. Nevertheless, with advances in statistical analytic procedures, this is a particularly exciting area of study, as researchers may now be able to better understand within-person relations of impulsivity and problematic alcohol use (see Lievens [100] for a recent review discussing personality-situation interplay and assessment approaches to broaden the range of methodological techniques in personality research). As discussed, we suggest that a unifying conceptualization, consistent nomenclature, state- and trait-level assessment, and EMA designs may be particularly useful in elucidating precise relations between impulsive dispositions and alcohol.

## Abbreviations

AUD: Alcohol use disorder
AUDIT: Alcohol use disorder identification test
BIS-11: Barrett impulsiveness scale – 11^th^ revision
CPT: Continuous performance test
DMT: Delayed memory test
DOSPERT: Domain-specific risk-taking scale
DSM-5: Diagnostic and statistical manual of mental disorders, 5^th^ edition
EFA: Exploratory factor analyses
EMA: Ecological momentary assessment
EPQ-R: Eysenck personality questionnaire-revised
IMT: Immediate memory test
MCQ: Monetary choice questionnaire
MIS: Momentary impulsivity scale
MPQ: Multidimensional personality questionnaire
MTMM: Multi-trait-multimethod
SCRAM: Secure continuous remote alcohol monitor
SKIP: Single key impulsivity paradigm
SSRT: Stop-signal reaction time task
TCIP: Two choice impulsivity paradigm
TPQ: Tridimensional personality questionnaire

## References

[1] American Psychiatric Association (2013). Diagnostic and statistical manual of mental disorders.

[2] Lejuez CW, Magidson JF, Mitchell SH, Sinha R, Stevens MC, De Wit H (2010). Behavioral and biological indicators of impulsivity in the development of alcohol use, problems, and disorders. Alcohol Clin Exp Res.

[3] Sher KJ, Trull TJ, Bartholow B, Vieth A, Bland H, Leonard K (1999). Personality and alcoholism: Issues, methods, and etiological processes. Psychological theories of drinking and alcoholism.

[4] Smith GT, Fischer S, Cyders MA, Annus AM, Spillane NS, McCarthy DM (2007). On the validity and utility of discriminating among impulsivity-like traits. Assessment.

[5] Littlefield AK, Sher KJ, Sher KJ (2016). Personality and substance use disorders. Handbook of substance use and substance use disorders.

[6] Berg JM, Latzman RD, Bliwise NG, Lilienfeld SO (2015). Parsing the heterogeneity of impulsivity: a meta-analytic review of the behavioral implications of the UPPS for psychopathology. Psychol Assess.

[7] Sharma L, Markon KE, Clark LA (2014). Toward a theory of distinct types of “impulsive” behaviors: A meta-analysis of self-report and behavioral measures. Psychol Bull.

[8] Evenden J (1999). Impulsivity: a discussion of clinical and experimental findings. J Psychopharmocol.

[9] Cyders MA (2015). The misnomer of impulsivity: Commentary on “choice impulsivity” and “rapid-response impulsivity” articles by Hamilton and colleagues. Personal Disord.

[10] Block J (1995). A contrarian view of the five-factor approach to personality description. Psychol Bull.

[11] Tellegen A, Tuma AH, Maser JD (1985). Structure of mood and personality and their relevance to assessing anxiety, with an emphasis on self-report. Anxiety and the anxiety disorders.

[12] Eysenck SBG, Eysenck HJ, Barrett P (1985). A revised version of the psychoticism scale. Pers Individ Differ.

[13] Zuckerman M, Kuhlman D, Joireman J, Teta P, Kraft M (1993). A comparison of the three structural models for the personality: the big three, the big five and the alternative five. J Pers Soc Psychol.

[14] Smith GT, McCarthy DM, Zapolski TCB (2009). On the value of homogeneous constructs for construct validation, theory testing, and the description of psychopathology. Psychol Assess.

[15] Blanchard BE, Stevens AK, Littlefield AK, Talley AE, Brown JL (2017). Examining the link between nonmedical use of sedatives, tranquilizers, and pain relievers with dispositions toward impulsivity among college students. Addict Behav.

[16] Tomko RL, Solhan MB, Carpenter RW, Brown WC, Jahng S, Wood PK (2014). Measuring impulsivity in daily life: the momentary impulsivity scale. Psychol Assess.

[17] Sharma L, Kohl K, Morgan TA, Clark LA (2013). “Impulsivity”: relations between self-report and behavior. J Pers Soc Psychol.

[18] Reise SP, Moore TM, Sabb FW, Brown AK, London ED (2013). The Barratt Impulsiveness Scale–11: reassessment of its structure in a community sample. Psychol Assess.

[19] Patton JH, Stanford MS, Barratt ES (1995). Factor structure of the Barratt impulsiveness scale. J Clin Psychol.

[20] Lynam D, Smith GT, Cyders MA, Fischer S, Whiteside SA. The UPPS-P: a multidimensional measure of risk for impulsive behavior. Unpublished technical report. 2007;

[21] Stanford MS, Mathias CW, Dougherty DM, Lake SL, Anderson NE, Patton JH (2009). Fifty years of the Barratt Impulsiveness Scale: An update and review. Pers Indiv Differ.

[22] Cyders MA, Smith GT (2008). Emotion-based dispositions to rash action: positive and negative urgency. Psychol Bull.

[23] Whiteside SP, Lynam DR (2001). The five factor model and impulsivity: using a structural model of personality to understand impulsivity. Pers Individ Differ.

[24] Weafer J, Baggott MJ, de Wit H (2013). Test–retest reliability of behavioral measures of impulsive choice, impulsive action, and inattention. Exp Clin Psychopharmacol.

[25] Stevens AK, Blanchard BE, Shi M, Littlefield AK. Testing measurement invariance of the UPPS-P Impulsive Behavior Scale in Hispanic/Latino and non-Hispanic/Latino college students. Psychological Assessment. 2017; 10.1037/pas0000494. 10.1037/pas0000494 28481578

[26] Cyders MA (2013). Impulsivity and the sexes: measurement and structural invariance of the UPPS-P Impulsive Behavior Scale. Assessment.

[27] Hamilton CM, Strader LC, Pratt JG, Maiese D, Hendershot T, Kwok RK (2011). The PhenX Toolkit: get the most from your measures. Am J Epidemiol.

[28] Cyders MA, Coskunpinar A (2011). Measurement of constructs using self-report and behavioral lab tasks: Is there overlap in nomothetic span and construct representation for impulsivity?. Clin Psychol Rev.

[29] Sperry SH, Lynam DR, Walsh MA, Horton LE, Kwapil TR (2016). Examining the multidimensional structure of impulsivity in daily life. Pers Individ Differ.

[30] Northrup DA. The problem of the self-report in survey research. North York, Ontario, Canada: Institute for. Social Research. 1997;

[31] Dick DM, Smith G, Olausson P, Mitchell SH, Leeman RF, O'Malley SS (2010). Understanding the construct of impulsivity and its relationship to alcohol use disorders. Addict Biol.

[32] Hamilton KR, Littlefield AK, Anastasio N, Cunningham KA, Fink LA, Wing VC, et al. Rapid-response impulsivity: Definitions, measurement issues, and clinical implications. Pers Disord: Theory, Res Treatment. 2015;6:168–81. 10.1037/per0000100 PMC4476624 25867840

[33] Hamilton KR, Mitchell MR, Wing VC, Balodis IM, Bickel WK, Filmore M, et al. Choice impulsivity: Definitions, measurement issues, and clinical implications. Personality Disorders: Theory. Res Treatment. 2015;6:182–98. 10.1037/per0000099 PMC4535726 25867841

[34] Dougherty DM, Mathias CW, Marsh DM, Jagar AA (2005). Laboratory behavioral measures of impulsivity. Behav Res Methods.

[35] Dougherty DM, Marsh DM, Mathias CW (2002). Immediate and delayed memory tasks: a computerized behavioral measure of memory, attention, and impulsivity. Behav Res Methods.

[36] Conners KC, Staff MHS. Conners’ continuous performance test II. North Tonawanda. NY: Multi-Health Systems.

[37] Kirby KN, Petry NM, Bickel WK (1999). Heroin addicts have higher discount rates for delayed rewards than non-drug-using controls. J Exp Psychol Gen.

[38] Fillmore MT, Weafer J. In: MacKillop J, de Wit H, editors. Behavioral inhibition and addiction. West Sussex: Wiley; 2013, p. 135–67.

[39] MacKillop J, Weafer J, C Gray J, Oshri A, Palmer A, de Wit H (2016). The latent structure of impulsivity: impulsive choice, impulsive action, and impulsive personality traits. Psychopharmacology.

[40] Cyders MA, Coskunpinar A (2012). The relationship between self-report and lab task conceptualizations of impulsivity. J Res Pers.

[41] Stevens AK, Littlefield AK, Talley AE, Brown JL (2017). Do individuals higher in impulsivity drink more impulsively? A pilot study within a high risk sample of young adults. Addict Behav.

[42] Burris JL, Riley E, Puleo GE, Smith GT (2017). A longitudinal study of the reciprocal relationship between ever smoking and urgency in early adolescence. Drug Alcohol Depend.

[43] Riley EN, Rukavina M, Smith GT (2016). The reciprocal predictive relationship between high-risk personality and drinking: an 8-wave longitudinal study in early adolescents. J Abnorm Psychol.

[44] Smith GT, Guller L, Zapolski TC (2013). A comparison of two models of urgency: urgency predicts both rash action and depression in youth. Clin Psychol Sci.

[45] Smith GT, Cyders MA (2016). Integrating affect and impulsivity: The role of positive and negative urgency in substance use risk. Drug Alcohol Depend.

[46] Gullo MJ, Loxton NJ, Dawe S (2014). Impulsivity: four ways five factors are not basic to addiction. Addict Behav.

[47] Derefinko K, DeWall CN, Metze AV, Walsh EC, Lynam DR (2011). Do different facets of impulsivity predict different types of aggression?. Aggressive Behavior.

[48] Cloninger C (1987). Neurogenetic adaptive mechanisms. Science.

[49] Slutske WS, Heath AC, Madden PA, Bucholz KK, Statham DJ, Martin NG (2002). Personality and the genetic risk for alcohol dependence. J Abnorm Psychol.

[50] King KM, Patock-Peckham JA, Dager AD, Thimm K, Gates JR (2014). On the mismeasurement of impulsivity: trait, behavioral, and neural models in alcohol research among adolescents and young adults. Current Addiction Reports.

[51] Lansbergen MM, Schutter DJ, Kenemans JL (2007). Subjective impulsivity and baseline EEG in relation to stopping performance. Brain Res.

[52] Ramautar JR, Kok A, Ridderinkhof KR (2004). Effects of stop-signal probability in the stop-signal paradigm: the N2/P3 complex further validated. Brain Cogn.

[53] Ramautar JR, Slagter HA, Kok A, Ridderinkhof KR (2006). Probability effects in the stop-signal paradigm: the insula and the significance of failed inhibition. Brain Res.

[54] Rey-Mermet A, Gade M, Oberauer K. Should we stop thinking about inhibition? Searching for individual and age differences in inhibition as a psychometric construct. J Exp Psychol Learn Mem Cogn. 2017; 10.1037/xlm0000450. 10.1037/xlm0000450 28956944

[55] Hedge C, Powell G, Sumner P. The reliability paradox: why robust cognitive tasks do not produce reliable individual differences. Behav Res Methods. 2017; 10.3758/s13428-017-0935-1. 10.3758/s13428-017-0935-1 PMC5990556 28726177

[56] Van de Mortel TF (2008). Faking it: social desirability response bias in self-report research. The Australian Journal of Advanced Nursing.

[57] Campbell DT, Fiske DW (1959). Convergent and discriminant validation by the multitrait-multimethod matrix. Psychol Bull.

[58] Hasin DS, O’Brien CP, Auriacombe M, Borges G, Bucholz K, Budney A (2013). DSM-5 criteria for substance use disorders: recommendations and rationale. Am J Psychiatry.

[59] Lane SP, Sher KJ (2015). Limits of current approaches to diagnosis severity based on criterion counts: an example with *DSM-5* alcohol use disorder. Clin Psychol Sci.

[60] Martin CS, Chung T, Kirisci L, Langenbucher JW (2006). Item response theory analysis of diagnostic criteria for alcohol and cannabis use disorders in adolescents: implications for DSM-V. J Abnorm Psychol.

[61] Lane SP, Steinley D, Sher KJ (2016). Meta-analysis of DSM alcohol use disorder criteria severities: structural consistency is only ‘skin deep’. Psychol Med.

[62] Sobell LC, Sobell MB, Allen JP, Columbus M (1994). Alcohol consumption measures. Assessing alcohol problems.

[63] Williams GD, Proudfit AH, Quinn EA, Campbell KE (1994). Variations in quantity-frequency measures of alcohol consumption from a general population survey. Addiction.

[64] Agrawal S, Sobell MB, Sobell LC, Belli RF, Stafford FP, Alwin DF (2008). The Timeline Followback: a scientifically and clinically useful tool for assessing substance use. Calendar and time diary methods in life course research.

[65] Collins RL, Parks GA, Marlatt GA (1985). Social determinants of alcohol consumption: The effects of social interaction and model status on the self-administration of alcohol. J Consult Clin Psychol.

[66] Babor TF, Higgins-Biddle JC, Saunders JB, Monteiroo MGAUDIT. The Alcohol Use Disorders Identification Test: guidelines for use in primary care. 2^nd^ ed. Geneva: World Health. Organization. 2001;

[67] Allen JP, Litten RZ, Fertig JB, Babor T (1997). A review of research on the Alcohol Use Disorders Identification Test (AUDIT). Alcohol Clin Exp Res.

[68] de Meneses-Gaya C, Zuardi AW, Loureiro SR, Crippa JAS. Alcohol Use Disorders Identification Test (AUDIT): an updated systematic review of psychometric properties. Psychol Neurosci. 2009;2:83–97.

[69] Read JP, Merrill JE, Kahler CW, Strong DR (2007). Predicting functional outcomes among college drinkers: reliability and predictive validity of the Young Adult Alcohol Consequences Questionnaire. Addict Behav.

[70] Hurlbut SC, Sher KJ (1992). Assessing alcohol problems in college students. Journal of American College Health.

[71] Neal DJ, Corbin WR, Fromme K (2006). Measurement of alcohol-related consequences among high school and college students: application of item response models to the Rutgers Alcohol Problem Index. Psychological Assessment.

[72] White HR, Labouvie EW (1989). Towards the assessment of adolescent problem drinking. J Stud Alcohol.

[73] Pearson MR, Kite BA, Henson JM (2012). Unique direct and indirect effects of impulsivity-like traits on alcohol-related outcomes via protective behavioral strategies. J Drug Educ.

[74] Mallett KA, Varvil-Weld L, Borsari B, Read JP, Neighbors C, White HR (2013). An update of research examining college student alcohol-related consequences: new perspectives and implications for interventions. Alcohol Clin Exp Res.

[75] Littlefield AK, Stevens AK, Sher KJ (2014). Impulsivity and alcohol involvement: multiple, distinct constructs and processes. Current Addiction Reports.

[76] Littlefield AK, Sher KJ, Wood PK (2009). Is “maturing out” of problematic alcohol involvement related to personality change?. J Abnorm Psychol.

[77] Henges AL, Marczinski CA (2012). Impulsivity and alcohol consumption in young social drinkers. Addict Behav.

[78] Papachristou H, Nederkoorn C, Havermans R, van der Horst M, Jansen A (2012). Can’t stop the craving: the effect of impulsivity on cue-elicited craving for alcohol in heavy and light social drinkers. Psychopharmacology.

[79] Bjork JM, Hommer DW, Grant SJ, Danube C (2004). Impulsivity in abstinent alcohol-dependent patients: Relation to control subjects and type 1–/type 2–like traits. Alcohol.

[80] Evren C, Durkaya M, Evren B, Dalbudak E, Cetin R (2012). Relationship of relapse with impulsivity, novelty seeking and craving in male alcohol-dependent inpatients. Drug Alcohol Rev.

[81] Rubio G, Jiménez M, Rodríguez-Jiménez R, Martínez I, Ávila C, Ferre F (2008). The role of behavioral impulsivity in the development of alcohol dependence: a 4-year follow-up study. Alcohol Clin Exp Res.

[82] Dom G, D’haene P, Hulstijn W, Sabbe BGCC (2006). Impulsivity in abstinent early-and late-onset alcoholics: differences in self-report measures and a discounting task. Addiction.

[83] Dom G, Hulstijn W, Sabbe BGCC (2006). Differences in impulsivity and sensation seeking between early-and late-onset alcoholics. Addict Behav.

[84] Balodis IM, Potenza MN, Olmstead MC (2009). Binge drinking in undergraduates: relationships with gender, drinking behaviors, impulsivity and the perceived effects of alcohol. Behav Pharmacol.

[85] Coskunpinar A, Dir AL, Cyders MA (2013). Multidimensionality in impulsivity and alcohol Use: a meta-analysis using the UPPS model of impulsivity. Alcohol Clin Exp Res.

[86] Trull TJ, Wycoff AM, Lane SP, Carpenter RW, Brown WC (2016). Cannabis and alcohol use, affect and impulsivity in psychiatric out-patients' daily lives. Addiction.

[87] Dougherty DM, Mathias CW, Tester ML, Marsh DM (2004). Age at first drink relates to behavioral measures of impulsivity: the immediate and delayed memory tasks. Alcohol Clin Exp Res.

[88] Dougherty DM, Bjork JM, Marsh DM, Moeller GF (2000). A comparison between adults with conduct disorder and normal controls on a continuous performance test: differences in impulsive response characteristics. Psychol Rec.

[89] Kollins SH (2003). Delay discounting is associated with substance use in college students. Addict Behav.

[90] Ortner CN, MacDonald TK, Olmstead MC (2003). Alcohol intoxication reduces impulsivity in the delay-discounting paradigm. Alcohol Alcohol.

[91] Dougherty DM, Marsh-Richard DM, Hatzis ES, Nouvion SO, Mathias CW (2008). A test of alcohol dose effects on multiple behavioral measures of impulsivity. Drug Alcohol Depend.

[92] Weafer J, Fillmore MT (2016). Low-dose alcohol effects on measures of inhibitory control, delay discounting, and risk-taking. Current Addiction Reports.

[93] Meda SA, Stevens MC, Potenza MN, Pittman B, Andrews MM, Thomas AD (2009). Investigating the behavioral and self-report constructs of impulsivity domains using principal component analysis. Behav Pharmacol.

[94] Stephan RA, Alhassoon OM, Allen KE, Wollman SC, Hall M, Thomas WJ (2017). Meta-analyses of clinical neuropsychological tests of executive dysfunction and impulsivity in alcohol use disorder. Am J Drug Alcohol Abuse.

[95] Blais AR, Weber EUA (2006). domain-specific risk-taking (DOSPERT) scale for adult populations. Judgm Decis Mak.

[96] Trull TJ, Ebner-Priemer UW (2009). Using Experience Sampling Methods/Ecological Momentary Assessment (ESM/EMA) in clinical assessment and clinical research: introduction to the special section. Psychological Assessment.

[97] Dougherty DM, Hill-Kapturczak N, Liang Y, Karns TE, Lake SL, Cates SE, Roache JD (2015). The potential clinical utility of transdermal alcohol monitoring data to estimate the number of alcoholic drinks consumed. Addict Disord Their Treat.

[98] Nahum-Shani I, Hekler EB, Spruijt-Metz D (2015). Building health behavior models to guide the development of just-in-time adaptive interventions: a pragmatic framework. Health Psychol.

[99] Durbin CE, Hicks BM (2017). To learn something new, try something different. Eur J Pers.

[100] Lievens F (2017). Assessing personality-situation interplay in personnel selection: toward more integration into personality research. Eur J Pers.

